# Longitudinal Analysis of Antibody Responses to the mRNA BNT162b2 Vaccine in Patients Undergoing Maintenance Hemodialysis: A 6-Month Follow-Up

**DOI:** 10.3389/fmed.2021.796676

**Published:** 2021-12-24

**Authors:** André Weigert, Marie-Louise Bergman, Lígia A. Gonçalves, Iolanda Godinho, Nádia Duarte, Rita Abrantes, Patrícia Borges, Ana Brennand, Vanessa Malheiro, Paula Matoso, Onome Akpogheneta, Lindsay Kosack, Pedro Cruz, Estela Nogueira, Magda Pereira, Ana Ferreira, Marco Marques, Telmo Nunes, João Faro-Viana, Jocelyne Demengeot, Carlos Penha-Gonçalves

**Affiliations:** ^1^DaVita Óbidos, Óbidos, Portugal; ^2^Serviço de Nefrologia, Centro Hospitalar de Lisboa Ocidental, Hospital Santa Cruz, Carnaxide, Portugal; ^3^Faculdade de Medicina, Instituto de Farmacologia e Neurociências, Universidade de Lisboa, Lisboa, Portugal; ^4^IGC, Instituto Gulbenkian de Ciência, Oeiras, Portugal; ^5^Serviço de Nefrologia e Transplantação Renal, Centro Hospitalar de Lisboa Norte EPE, Lisboa, Portugal; ^6^Serviço de Nefrologia, Centro Hospitalar do Médio Tejo, Torres Novas, Portugal; ^7^Serviço de Nefrologia, Hospital Das Forças Armadas, Lisboa, Portugal; ^8^Affidea Laboratório Lisboa, Lisboa, Portugal; ^9^CIISA, Centro de Investigação Interdisciplinar em Sanidade Animal, Faculdade de Medicina Veterinária, Universidade de Lisboa, Lisboa, Portugal; ^10^Serviço de Patologia Clínica, Centro Hospitalar de Lisboa Ocidental EPE, Carnaxide, Portugal

**Keywords:** BNT162b2, chronic hemodialysis, COVID-19, IgG, SARS-CoV-2, vaccine

## Abstract

**Background:** Patients on hemodialysis (HD) are at higher risk for COVID-19, overall are poor responders to vaccines, and were prioritized in the Portuguese vaccination campaign.

**Objective:** This work aimed at evaluating in HD patients the immunogenicity of BTN162b2 after the two doses induction phase, the persistence of specific antibodies along time, and factors predicting these outcomes.

**Methods:** We performed a prospective, 6-month long longitudinal cohort analysis of 156 HD patients scheduled to receive BTN162b2. ELISA quantified anti-spike IgG, IgM, and IgA levels in sera were collected every 3 weeks during the induction phase (t0 before vaccine; t1, d21 post first dose; and t2 d21 post second dose), and every 3–4 months during the waning phase (t3, d140, and t4, d180 post first dose). The age-matched control cohort was similarly analyzed from t0 to t2.

**Results:** Upon exclusion of participants identified as previously exposed to SARS-CoV-2, seroconversion at t1 was lower in patients than controls (29 and 50%, respectively, *p* = 0.0014), while the second vaccine dose served as a boost in both cohorts (91 and 95% positivity, respectively, at t2, *p* = 0.2463). Lower response in patients than controls at t1 was a singularity of the participants ≤ 70 years (*p* = 2.01 × 10^−05^), associated with immunosuppressive therapies (*p* = 0.013), but not with lack of responsiveness to hepatitis B. Anti-spike IgG, IgM, and IgA levels decreased at t3, with IgG levels further waning at t4 and resulting in >30% seronegativity. Anti-spike IgG levels at t1 and t4 were correlated (ρ = 0.65, *p* < 2.2 × 10^−16^).

**Conclusions:** While most HD patients seroconvert upon 2 doses of BNT162b2 vaccination, anti-spike antibodies levels wane over the following 4 months, leading to early seroreversion in a sizeable fraction of the patients. These findings warrant close monitoring of COVID-19 infection in vaccinated HD patients, and advocate for further studies following reinforced vaccination schedules.

## Introduction

Patients with chronic kidney disease requiring renal replacement therapy and receiving in-center hemodialysis (HD) treatment are at an increased risk of SARS-Cov-2 infection, and of severe COVID-19 (1). Moreover, HD patients may pose additional stress in the hospital dialysis capacity when admitted, as most receive routine dialysis treatments as outpatients.

End-stage renal disease is simultaneously associated with systemic inflammation (2) and immune deficiency (3). Systemic inflammation contributes to atherosclerosis, cardiovascular disease, cachexia, and anemia, contributing to enhanced susceptibility to severe COVID, whereas immune deficiency leads to impaired response to vaccination and increased incidence and severity of microbial infections. Several studies evidenced abnormal immune response both to viral infection and to vaccination in HD patients (4–6). Blunted antibody responses to influenza (7), pneumococcal (8), and hepatitis B vaccination (9) are indicators of abnormal adaptative immunity in these patients. This lack of response is in part due to uremic toxins that may lead to alterations in B-lymphocyte function, among others (10). Kidney deficiency is associated with vitamin D insufficiency contributing to weakened immunity. Given the impaired antibody response of HD patients to other vaccines, there are concerns regarding the robustness and durability of the humoral response induced by SARS-CoV-2 vaccines in this population.

All patients undergoing HD (about 12,000 in Portugal) received 2 doses of the Pfizer-BioNTech mRNA BNT162b2 vaccine 3 weeks apart, according to the manufacturer's and health authority's recommendations, in January–February 2021. The third dose of vaccination for elderly people, including dialysis patients, was approved in October 2021, a date posterior to the present study. Other SARS-CoV-2 vaccines distributed in Portugal (Moderna mRNA-1273, the vectorial Oxford/AstraZeneca-AZD1222, and Janssen-Ad26.COV2.S) were not administrated to HD patients.

In the general population, as evidenced in the 2–3 months follow-up of large-scale cohorts of reference health care workers (HCW), the 2-dose regimen of BNT162b2 is highly immunogenic and confers robust protection to COVID-19 and SARS-CoV-2 infection (11–13). In HD patients, initial studies revealed success in antibody generation, but reduced titers in comparison with healthy controls (14–16). Assessing the effectiveness of BNT162b2 in reducing infection, transmission, and severe disease requires very large cohorts, which for HD patients would require multicenter analysis. Hence, for SARS-CoV-2 as for other vaccines (above), antibodies could be used as proxy/biomarkers of vaccine immunity.

In this study, we aimed to evaluate the immunogenicity of mRNA BTN162b2 during the induction phase, the persistence, and decline of specific antibodies up to 6 months after initiation of the vaccination, and factors predicting these outcomes in patients undergoing HD.

## Materials and Methods

### Ethics Statement

This study was reviewed and approved by the Ethics committees of DaVita in Portugal (date 2021/03/06), Centro Hospitalar Lisboa Ocidental (Reference 2102, date 2021/01/12), and the Administração Regional de Saude Lisboa e Vale do Tejo (Reference 2105/CES/2021-date 2021/03/22) in compliance with the 1975 Declaration of Helsinki, as revised in 2013, and follows the international and national guidelines for health data protection. All participants provided their written informed consent to participate in the study.

### Study Design

**Figure 1:**
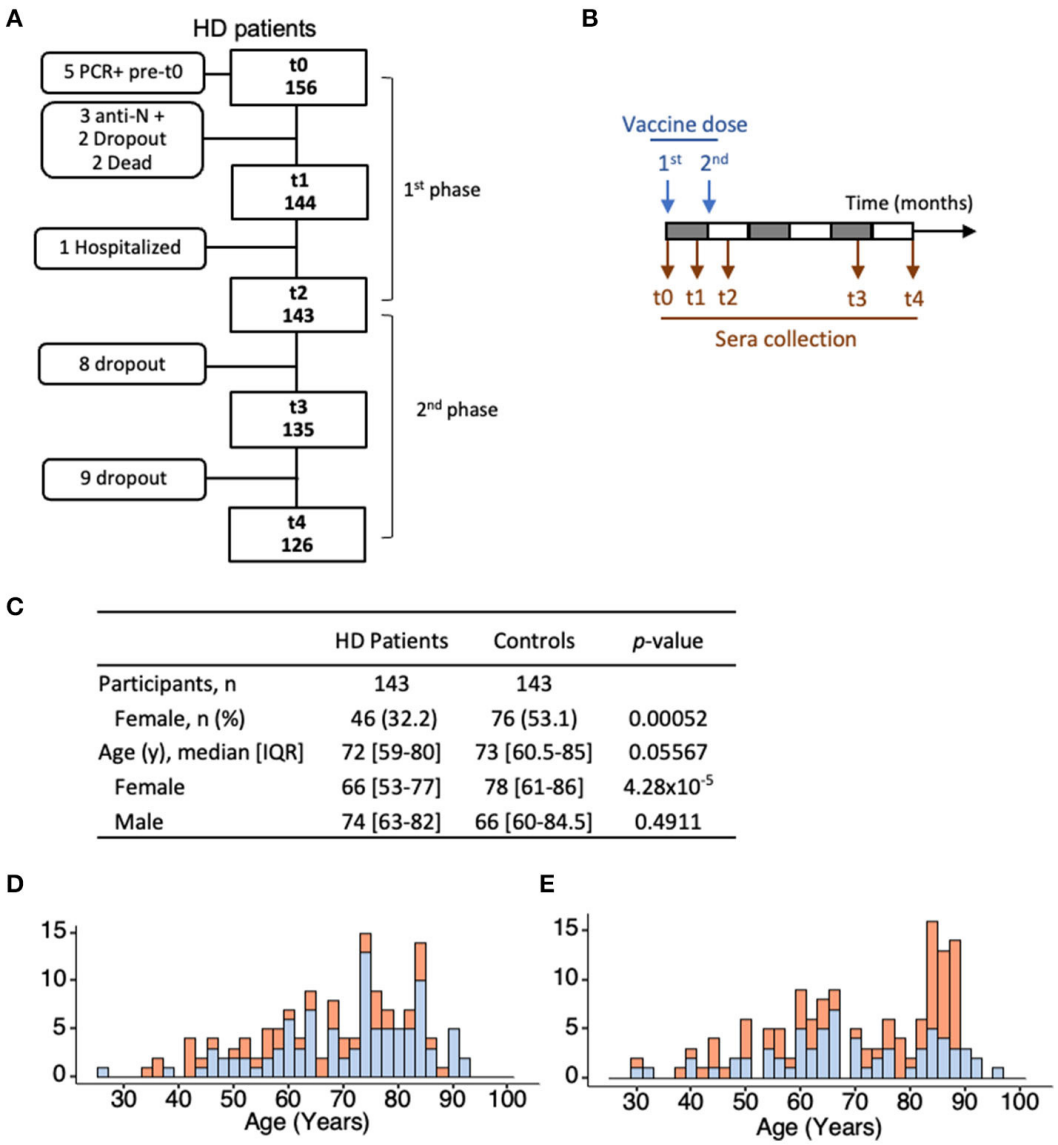
Patient and control cohorts. **(A)** Enrolment and funneling of HD patients during the first and second phases of the study, showing concordance to the study design (straight boxes), and exclusion criteria to the antibody analysis, dropouts, and death (rounded boxes). **(B)** Serum collections were performed at the time of inoculation of the first dose (t0); 21 days post-first dose (t1) and 42 days post-first dose (t2), and thereafter, at t3 (140 days post-first vaccine dose) and t4 (180 days post-first vaccine dose). **(C–E)** Age and sex profiles of the 143 patients and 143 controls analyzed for the first phase of the study. Differences in age and sex distribution between the two cohorts were evaluated using the Wilcoxon rank sum test (with continuity correction) and Pearson's Chi-squared test (with Yates' continuity correction), respectively. HD, hemodialyzed patients; *n*, number of individuals with a given event; IQR, interquartile range.

Patients were recruited using a non-probabilistic method by convenience and volunteer sampling. The study design was planned for a universe of 170 patients, based on the number of outpatients at the participating HD center. The study enrolled 156 patients with stage 5 chronic kidney disease (CKD) undergoing renal replacement therapy as outpatients at a single HD clinic (DaVita, Eurodial) in Óbidos, Portugal. An age-matched control cohort, without kidney disease, comprised 143 individuals selected from a larger cohort of 1,245 HCW and 146 nursing home residents (17). The effect size was calculated based on the Cohen's h method to establish the power analysis, which denoted that to detect a difference of 25% with significance level of *p* < 0.05 and power analysis of 80%, we need around *n* = 50 in each group. The group ≤ 70 years and >70 years are *n* = 66 and *n* = 77, respectively. Stratification by age range (27–70) years and (71–93) years, splits both patient and control cohorts equally in *n* = 66 and *n* = 77 participants, in the respective age category. All patient and control participants initiated BNT162b2 mRNA vaccination (Comirnaty^®^, Pfizer/BioNTech) according to the established schedule of 2 doses with a 3-week interval. For the first phase of the study (Immunogenicity), venous blood was collected on the day of the first vaccine dose (time 0, t0), 3 weeks later on the day of the second dose (t1), and 3 weeks after the second dose (t2). Participants with evidence of COVID-19 infection were excluded [serum reactivity against SARS-CoV-2 nucleocapsid (N) at time of enrolment (*n* = 3) or SARS-CoV-2 RNA positivity in RT-PCR test before enrolment (*n* = 2) or during the collection time (*n* = 3), in the patient cohort] (Figures 1A,B). The same selection was applied to the control cohort (17). Between t0 and t1, two patients died, and two patients dropped-out of the study. Between t1 and t2, one patient was hospitalized with a non-COVID-19 respiratory infection. For the second phase of the study (Antibody persistence), venous blood was collected from 126 patients at 140 days (t3) and 180 days (t4) post-first vaccine dose. In all cases, blood collections were performed before HD procedures were initiated. Patients who did not contribute to the 3 collection times of each study phase were excluded from the analysis. Clinical data were collected from medical records and a dedicated questionnaire.

### Antibody Measurements

The ELISA assay, used to quantify IgG, IgM, and IgA anti-full-length SARS-CoV-2 spike was adapted from (18), relies on antigen produced as in (19), was semiautomized to a 384-well-format and uses sera diluted at 1/50, according to a protocol to be detailed elsewhere. Assay performance was determined by testing 1,000 prepandemic sera and 40 COVID-19 patients diagnosed at least 10 days prior to sera collection. ROC curve analysis was determined at a specificity of 99.3, 99.2, and 99.2%, and a sensitivity of 95.9, 61.2, and 73.7% for IgG, IgM, and IgA, respectively. Individual assay readouts (OD values) were standardized using calibrators (pool of positive sera at predefined dilutions) and the normalized OD (ODnorm), adjusted to set ODnorm = 1 as the positivity cut-off for IgG, IgM, and IgA. Serial titration of 67 COVID-19 patients established that the assay is semiquantitative, has a dynamic range of 3 log titer, and with decreased discrimination power at ODnorm ≥ 1.8. Each sample was assayed in duplicates and any identified discrepancies were resolved by repeating the test. Antibodies against SARS-CoV-2 N antigen were measured by an electrochemiluminescence immunoassay from Roche Diagnostics (Elecsys^®^ Anti-SARS-CoV-2). Total IgG, IgM, and IgA at t2 were quantified using three immunoturbidimetric methods (PEG enhanced) from Siemens Healthineers, using Siemens Atellica CH Analyzer, following manufacturer's instructions.

### Statistical Analysis

Quade test was used to analyze individuals in temporal series, and Wilcoxon signed-rank test to analyze pairwise group comparisons between different time points, which includes the Benjamini-Hochberg (BH) method for *p-*value adjustment. The Wilcoxon rank sum test (Mann–Whitney *U*-Test) was used for pairwise comparison between age groups or for single time-point comparisons between control and patient groups. To test for the effect of clinical conditions, within a given group, on the magnitude of the antibody responses, the Wilcoxon rank sum test was used. All *p-*values in multiple comparisons were adjusted using the Benjamini-Hochberg (BH) method. Pearson's Chi-squared test (with Yates' continuity correction) was used to determine differences in Ig positivity between groups over time, and within groups at specific time points. Fisher's exact test was used to test for the effect of specific clinical parameters or treatments with categorical variables on Ig positivity when assumptions for the chi-squared test were not met. Correlation of Ig levels with clinical parameters was tested by linear regression using the Spearman correlation coefficient (ρ). All *p-*values were obtained with two-sided tests, at a significance level of 0.05. All statistical tests were carried out using established R scripts. For data management, graphical design, and statistical analysis we used R, version 4.0.4 GUI 1.74 and Rstudio version 1.1.463, and the main packages tidyverse, ggplot2, openxlsx, writexl, officer, rvg, and ggpubr (references in Supplementary Material). The text reports continuous variables as medians and interquartile ranges (IQR), and categorical variables are summarized using frequencies and percentages.

### Missing Data Management

Anti-spike antibodies measurements were performed on all participants who adhered to the study design (no missing data). For correlation analysis effects of clinical conditions, clinical parameters, or biometrics, whenever there were participants with variables not recorded, they were excluded from the analysis, and “*n*” is indicated in each figure and table.

## Results

### Cohort Characterization

This longitudinal prospective cohort study enrolled 156 patients on HD scheduled for BNT162b2 mRNA vaccination in January and February 2021 (Figure 1). A total of 143 participants, with no evidence of previous exposure to SARS-CoV-2 at the first collection (anti-N-antigen negative, no previous PCR tests positive), adhered to the three collection times of the first phase of the study addressing the vaccine immunogenicity. The median age was 72 years of age (y) [range (27–93), IQR (59–80)], and women represented 32% of the cohort. Eleven patients (8.8%) were under therapies potentially affecting immune responses, including corticosteroids (Table 1). The control cohort included 143 individuals with median age of 73 y [range (30–96), IQR (61–85)] and 53% women. For the second phase of the study addressing antibody persistence, 126/143 patients adhered to the additional two collection times.

**Table 1:** Clinical characterization of HD patients classified as non-responders or responders according to anti-spike IgG levels at t2.

**Characteristics**	**Non-responders**	**Responders**
	**(*N =* 13)**	**(*N =* 130)**
Sex, men, *n* (%)	9 (69.2)	88 (67.7)
Age (years), median [IQR]	86 [74–90]^*^	71 [59–79]^*^
Body Weight (Kg), median [IQR]	69 [56–74]	72 [63–83]
BMI (kg/m^2^), median [IQR]	24.7 [21.9–25.7]	26 [23–30]
Dialysis duration (months), median [IQR]	46 [30–116]	46 [20–113]
Kt/v, median [IQR]	1.8 [1.7–2.0]	1.7 [1.5–1.9]
**Laboratory parameters**		
Hemoglobin (g/dL), median [IQR]	11.7 [11.1–12.7]	11.1 [10.4–11.8]
Serum albumin (g/dL), median [IQR]	4.0[3.6–4.1]	4.0 [3.8–4.3]
Ferritin (ng/mL), median [IQR]	348 [238–520]	368 [230–527]
nPCR (g/kg/day), median [IQR]	0.94 [0.90–1.23]	1.11 [0.95–1.22]
CRP (mg/dL), median [IQR]	0.55 [0.20–2.81]	0.48 [0.15–1.25]
25(OH)D3 (ng/mL), median [IQR]	35.0 [29.9–48.6]	35.3 [26.0–45.0]
**Comorbidities**		
Age adjusted Charlson score, median [IQR]	8.0 [6.0–9.0]	7.0 [5.0–8.7]
Diabetes mellitus, *n* (%)	7 (53.8)	64 (49.2)
Cardiac disease (except essential hypertension) *n* (%)	7 (53.8)	55 (42.3)
Essential hypertension, *n* (%)	8 (61.5)	96 (73.8)
Congenital or acquired immunodeficiency, *n* (%)	–	6 (4.6)
Chronic pulmonary disease, *n* (%)	–	17 (13.1)
Chronic liver disease, *n* (%)	1 (7.7)	6 (4.6)
Rheumatic disease, *n* (%)	2 (15.4)	6 (4.6)
Cancer in the last 5 years (non-leukemia), *n* (%)	1 (7.7)	13 (10)
Tumor metastasis, *n* (%)	–	2 (1.5)
Leukemia, *n* (%)	2 (15.4)^†^	2 (1.5)^†^
Past Kidney transplant, *n* (%)	3 (23.1)	20 (15.4)
Kidney allograft still present, *n* (%)	3 (23.1)^‡^	7 (5.4)^‡^
**Medication**		
Erythropoiesis-stimulating agent, *n* (%)	8 (61.5)	101 (77.7)
Angiotensin-converting-enzyme inhibitor, *n* (%)	1 (7.7)	29 (22.3)
Statins, *n* (%)	6 (46.2)	66 (50.8)
Corticosteroid (Prednisolone 2.5–5mg/day), *n* (%)	3 (23.1)	8 (6.2)
Other immunossupressor/immunomodulator, *n* (%)	2 (15.4)	3 (2.3)
Tacrolimus, *n* (%)	1 (7.7)	2 (1.5)
Tacrolimus and Everolimus, *n* (%)	1 (7.7)	–
Hydroxychloroquine, *n* (%)	–	1 (0.8)
Non-steroidal anti-inflammatory drug, *n* (%)	2 (15.4)	9 (6.9)
Antithrombotic, *n* (%)	8 (61.5)	70 (53.8)
Antiviral, total *n* (%)	1 (7.7)	2 (1.5)
Aciclovir, *n* (%)	1 (7.7)	–
Abacavir, Lamivudine, Efavirenz, *n* (%)	–	1 (0.8)
Abacavir, Lamivudine, Raltegravir, *n* (%)	–	1 (0.8)
Ongoing Chemotherapy, *n* (%)	–	1 (0.8)
Anti-HBc positivity, *n* (%)	1 (7.7)	13 (10)
Anti-HBs positivity (>10 UI/L), *n* (%)	5 (38.5)	62 (47.7)
Anti-HBs positivity in anti-HBc negative, *n* (%)	4 (30.8)	50 (38.5)

*N, total number of individuals; n, number of individuals with a given event; t2, sera collected 42 days post-first vaccine dose; %, percentage; IQR, interquartile range; BMI, Body Mass Index; KT/V, measure of dialysis adequacy; nPCR, Normalized Protein Catabolic Rate; CRP, C-reactive protein; 25(OH)D3, calcifediol or vitamin D hydroxylated at the 25 Carbon; ESA, Erythropoiesis-stimulating agents; Anti-HBc, Hepatitis B core antigen antibodies; Anti-HBs, Hepatitis B surface antigen antibodies. Statistical tests to compare non-responders with responders were applied according to the type of variable (categorical—Fisher's exact test or continuous—Wilcoxon rank sum test)*.

* *W = 1,200, p-value = 0.0128; 95% CI [3.00–18.00];*

† *p-value = 0.0414;*

‡ *p-value = 0.0488; All others, not significant*.

### Antispike Antibody Response in the Induction Phase

**Figure 2:**
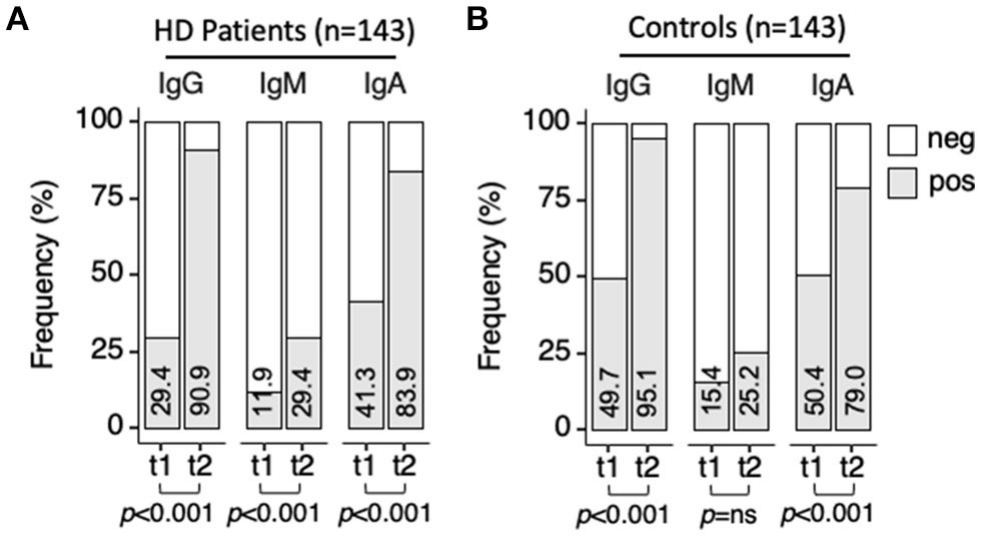
Anti-SARS-CoV-2-spike seroconversion upon vaccination. Sera collected at t1 and t2 were analyzed for anti-spike IgG, IgM, and IgA antibodies by ELISA. ODnorm≥1 was used as cut-off for positivity. Frequency of samples testing positive (gray bar) at t1 or t2 for each antibody class. **(A)** HD patients (*n* = 143). **(B)** controls (*n* = 143). Percentage of seroconversion indicated inside each bar. Pearson's Chi-squared test (with Yates' continuity correction) was used to determine differences over time in patients and controls (*p-*values indicated in the figure) and between patients and controls at t1, and t2 (*p-*values indicated in Supplementary Tables 1, 2). neg, negative; pos, positive; t1, sera collected 21 days post-first vaccine dose; t2, sera collected 42 days post-first vaccine dose.

Sera from HD patients and controls were analyzed for specific anti-SARS-CoV-2-spike antibodies (IgG, IgM, and IgA) using an ELISA calibrated with sera collected prior COVID-19 pandemic and from COVID-19 patients. We first analyzed seroconversion, discriminating positive/negative antibody reactivity (Figure 2, Supplementary Tables 1, 2). At t0, before vaccination, 100% of the control and 141/143 of the HD patients tested negative for anti-spike Ig. After a single vaccine dose (t1), seroconversion was lower in HD patients with only 42/143 (29.4%; 95%CI 22.5–37.3) patients developing anti-spike IgG antibodies when compared with 71/143 (49.7%; 95%CI 41.6–57.7) controls (patients vs. controls at t1, *p* = 0.0014). The second vaccine dose acted as a boost in both cohorts (t1 vs. t2, patients *p* = 8.01 × 10^−26^, controls *p* = 2.59 × 10^−17^), and both cohorts reached similar seropositivity rate (HD patients 130/143, 90.9%, 95% CI 85.1–94.6 and controls 136/143, 95.1%, 95%CI 90.2–97.6, *p* = 0.2463). Isotype class analysis of anti-spike antibodies revealed progression of IgA seroconversion in HD patients from t1 to t2 (41.3%; 95%CI 33.5–49.5 at t1, 83.9%; 95%CI 77.0–89.0 at t2, *p* = 2.27 × 10^−13^), reaching values similar to the control cohort (*p* = 0.3612). In contrast, prevalence of anti-spike IgM was low with modest increase along the vaccination schedule in both patients (11.9%; 95%CI 7.6–18.2 at t1 and 29.4%; 95%CI 22.5–37.3 at t2, *p* = 0.0005) and controls (15.4%, 95%CI 10.4–22.2 at t1 and 25.2%, 95%CI 18.8–32.9 at t2, *p* = 0.0559).

**Figure 3:**
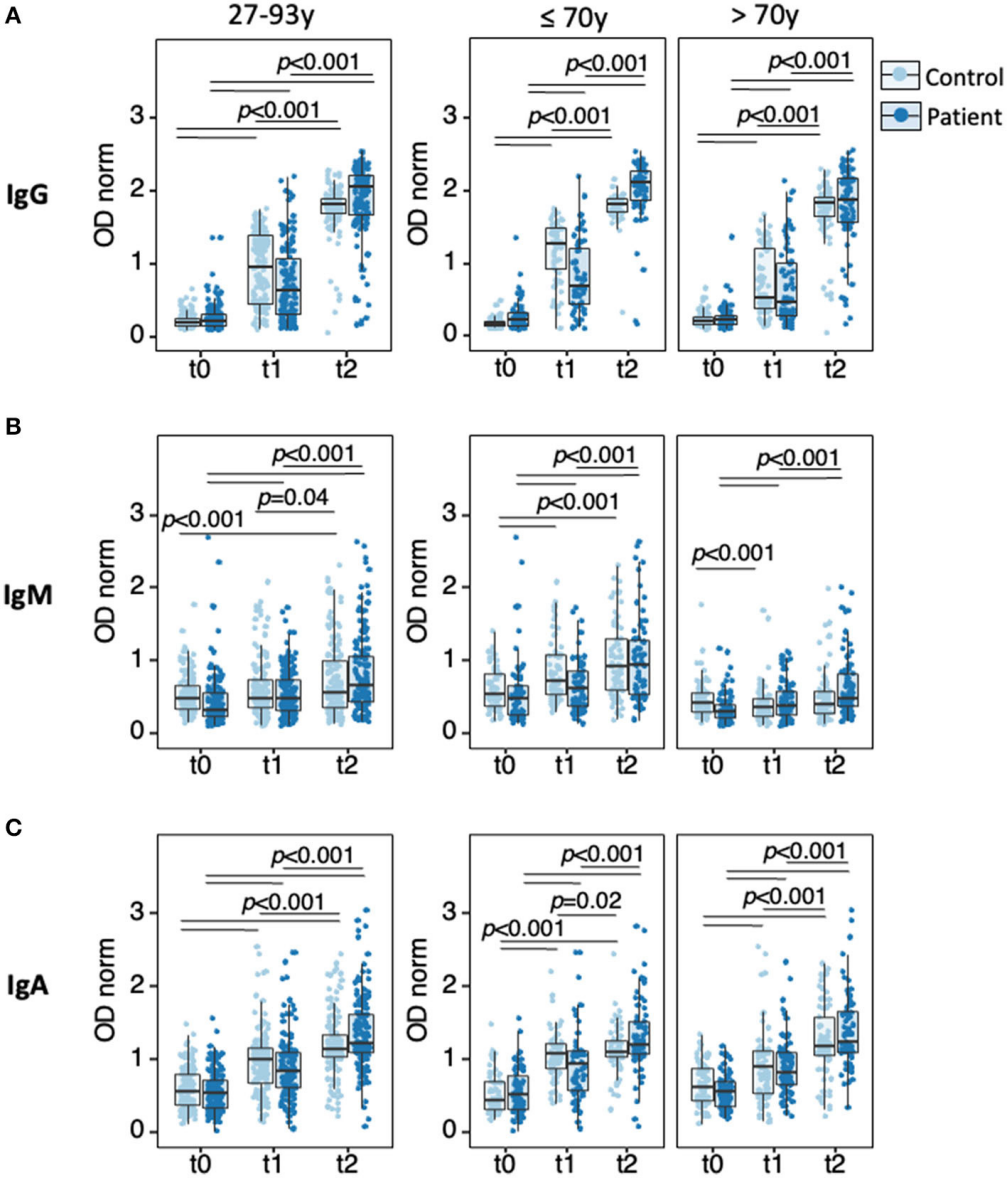
Anti-SARS-CoV-2-spike IgG, IgM, and IgA responses to vaccination. Sera collected at t0, t1, and t2 were analyzed by ELISA for semi-quantitative measurement of anti-spike IgG **(A)**, IgM **(B)**, and IgA **(C)** in HD patients (dark blue, *n* = 143) and age-matched controls (light blue, *n* = 143), in the full cohort (left panels) or upon stratification by age group (≤ 70 y, middle panels; >70 y, right panels). Data points represent individual subjects and are overlaid with boxes representing interquartile range (IQR), whiskers representing 1.5 IQR tails, and a central line representing median value. Differences were determined by Quade test for antibody levels along time in the full cohort (all 9 panels), Wilcoxon signed-rank test for pairwise comparison in each panel (*p-*values indicated by horizontal black bars), and Wilcoxon rank-sum to compare controls with patients at t1. Detailed *p-*values presented in Supplementary Tables 3–7. t0, sera collected on the day of first vaccine dose; t1, sera collected 21 days post-first vaccine dose; t2, sera collected 42 days post-first vaccine dose.

Semiquantitative analysis of antibody levels using normalized OD values (ODnorm) revealed significant increase of all three isotypes from t0 to t1, an effect of the first vaccine dose, and from t1 to t2, an effect of the second vaccine dose, in both patients and controls (Figure 3, Supplementary Tables 3–7). Within the patient cohort, most striking was the boosting effect of the second dose on anti-spike IgG levels (median [IQR]: 0.63 [0.32–1.08]) at t1; 2.05 [1.67–2.23] at t2, *p* = 2.2 × 10^−16^) and to a lower extent on anti-spike IgA levels (median [IQR]: (0.85 [0.63–1.10] at t1; 1.22 [1.10–1.63] at t2, *p* = 2.2 × 10^−16^), while anti-spike IgM was only modestly increased (0.49 [0.32–0.75] at t1; 0.66 [0.45–1.07] at t2, *p* = 4.5 × 10^−15^).

Comparison between patients and controls revealed lower anti-spike IgG levels in patients after the first vaccine dose (0.63 [0.32–1.08] in patients; 0.96 [0.46–1.39] in controls, *p* = 4.98 × 10^−04^), an effect not observed for the other isotypes.

To test whether the lower response of HD patients to the first vaccine dose holds across age, each cohort was partitioned in two age groups (right panels in Figure 3), ≤ 70 y (range (27–70), median 58, IQR [50–64), *n* = 66 for patients and range (30–70), median 60, IQR [51–64], *n* = 66 for controls) and >70 y (range (71–93), median 8, IQR [75–84], *n* = 77 for patients and range (71–96), median 85 IQR [70–88], *n* = 77 for controls). In controls and patients, elderly individuals presented similar low anti-spike IgG levels at t1, (0.47 [0.28–1.00] in patients; 0.52 [0.38–1.21] in controls, *p* = 0.2492). In contrast, in the ≤ 70 y groups, HD patients presented lower IgG response at t1 (0.68 [0.45–1.21] in patients, 1.27 [0.93–1.49] in controls, *p* = 2.01 × 10^−05^). In controls, age was clearly associated with lower IgG levels at t1 (*p* = 5.09 × 10^−07^), whereas in patients age effect was barely significant (*p* = 0.050). Together, these data indicate that the younger group contributed to most of the lower response observed at t1 when analyzing the full HD cohort. Similar analysis after the second vaccination dose could not be directly processed, as most samples in the control group reached values above the dynamic range of the assay.

### Factors Predicting Immunogenicity of BNT162B2 in HD Patients

**Figure 4:**
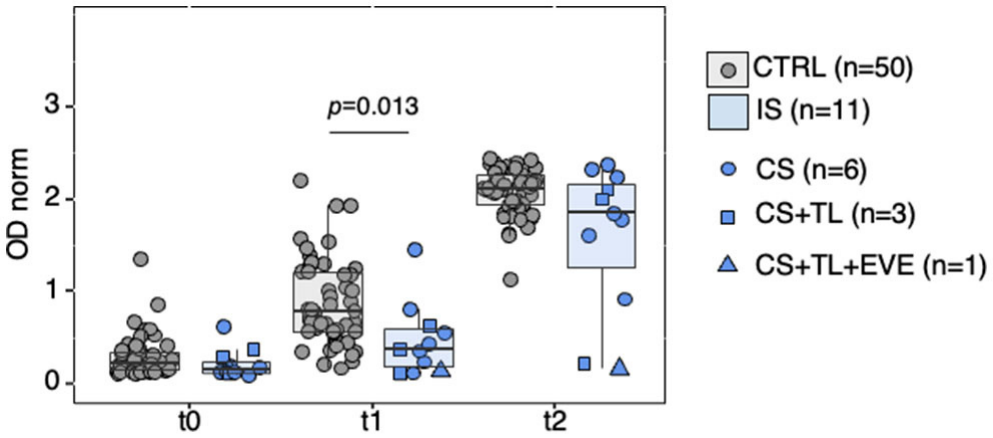
Anti-SARS-CoV-2-spike IgG reactivity in HD patients under immunosuppressive therapy. Partitioning of data presented in Figure 3, showing HD patients treated with immunosuppressive drugs (IS, *n* = 11) or not (CTRL, *n* = 50). Data points represent individual subjects and are overlaid with boxes representing interquartile range (IQR), whiskers representing 1.5 IQR tails, and a central line representing median value. At each time point differences in antibody levels between groups were determined with the Wilcoxon rank sum test (with continuity correction); significant *p-*values indicated in the figure. IS, immunosuppressive drugs; CTRL, controls; CS, corticosteroids; TL, Tacrolimus; EVE, Everolimus. Detailed *p-*values presented in Supplementary Table 9. t0, sera collected on the day of first vaccine dose; t1, sera collected 21 days post-first vaccine dose; t2, sera collected 42 days post-first vaccine dose.

The HD cohort encompassed 11 patients under immunosuppressive (IS) therapy (age range (42–63), median 56, IQR [51.0–58.0]). All IS patients received Prednisolone 2.5–5 mg/day, a mild IS regimen. Four patients were in addition treated with Tacrolimus, a strong IS drug (Table 1). Levels of anti-spike IgG antibodies in this small subgroup were compared with those of a subset of patients not on IS and selected from the full HD cohort (age range (42–69), median 60, IQR [53.0–64.0], *n* = 50) Figure 4, Supplementary Tables 8, 9). The levels of anti-spike IgG antibodies elicited by the first vaccine dose (t1), but not by the second dose, were lower in IS than control patients (*p* = 0.013 at t1). Of the 4 patients under Tacrolimus therapy, 2 were non-responders and 2 were responders at t2.

**Figure 5:**
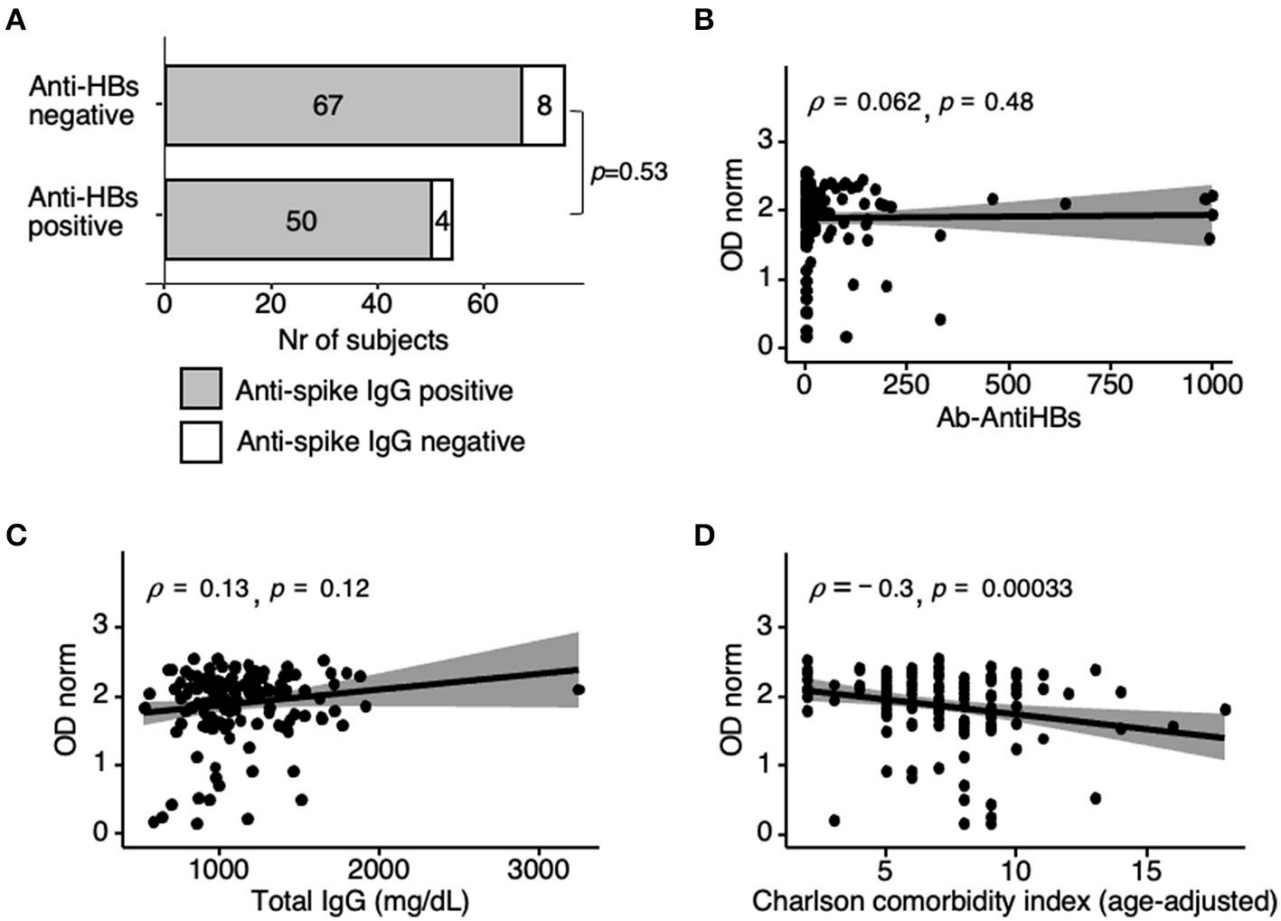
Correlation analysis of anti-spike IgG responses at t2. **(A)** Anti-spike positivity as in Figure 2, now in *n* = 75 non-responders and *n* = 54 responders to previous hepatitis B vaccination (anti-HBs antibody cut-off >10 mIU/ml). Fischer's test *p*-value = 0.53; OR = 1.49, 95%CI [0.48–4.65]. Fourteen anti-HBc reactive participants (i.e., previously infected with HBV) were excluded. **(B)** Spearman correlation coefficients (ρ) analysis of anti-spike IgG with anti-HBs antibody levels in anti HBc non-reactive individuals (*n* = 129, same as in **A**). **(C,D)** Spearman correlation coefficients (ρ) analysis of anti-spike IgG levels with total serum IgG also determined at t2 (*n* = 142) **(C)**, and with age-adjusted Charlson-comorbidity index (*n* = 142) **(D)**. Shaded areas represent 95% confidence interval. Differences were determined by Spearman's rank-order non-parametric test; t2, sera collected 42 days post-first vaccine dose.

Responsiveness to Hepatitis B vaccination is an indicator of immune competence. Anti-HBs antibody levels were available for 129 HD patients who tested negative for anti-HBc (hence, presumed not previously exposed to Hepatitis B virus). Of these, only 54/129 (42%) tested anti-HBs positive, whereas 117/129 (91%) were positive for anti-spike IgG at t2. Responsiveness to Hepatitis B and to BNT162b2 vaccine at t2 did not correlate, either measuring seroconversion (*p* = 0.53; OR = 1.49, 95% CI [0.48–4.65]) (Figure 5A) or specific antibodies levels (ρ = 0.062, *p* = 0.48) (Figure 5B). Likewise, neither total IgG levels nor lymphocyte counts measured for 142 patients at t2, correlated with anti-spike IgG levels (Figure 5C, Supplementary Figure 1).

We next analyzed indicators of kidney disease severity or activity. Neither time in dialysis nor levels of 25-hydroxycholecalciferol, C-reactive protein, hemoglobin, Ferritin, epoetin dosage, nor normalized protein catabolic rate significantly correlated with anti-spike IgG levels (Supplementary Figure 1).

Reviewing the clinical data of the 13/143 HD patients who remained anti-spike IgG negative at t2 was not informative due to the small sample size in each clinical category, and possible confounding factors due to multiple comorbidities (Table 1). Among these 13 non-responders, 3 were in their 50th, 3 in their 70th decade, and 7 were >84 y. In the control cohort, 7 participants were non-responders, 1 was 54 and under strong IS therapy, and 6 were >84 y. Altogether, these results support advanced age, and incidentally, immunosuppression is a *bona fide* factor affecting seroconversion in the general population, as for HD patients. Finally, and in concordance with multiple factors conditioning the amplitude of the humoral response to BNT162b2 vaccine, the age-adjusted Charlson comorbidity index was weakly inversely correlated with anti-spike IgG levels at t2 (ρ = −0.3, *p-*value = 0.0003) (Figure 5D).

### Persistence vs. Seroreversion of Antispike Responses

**Figure 6:**
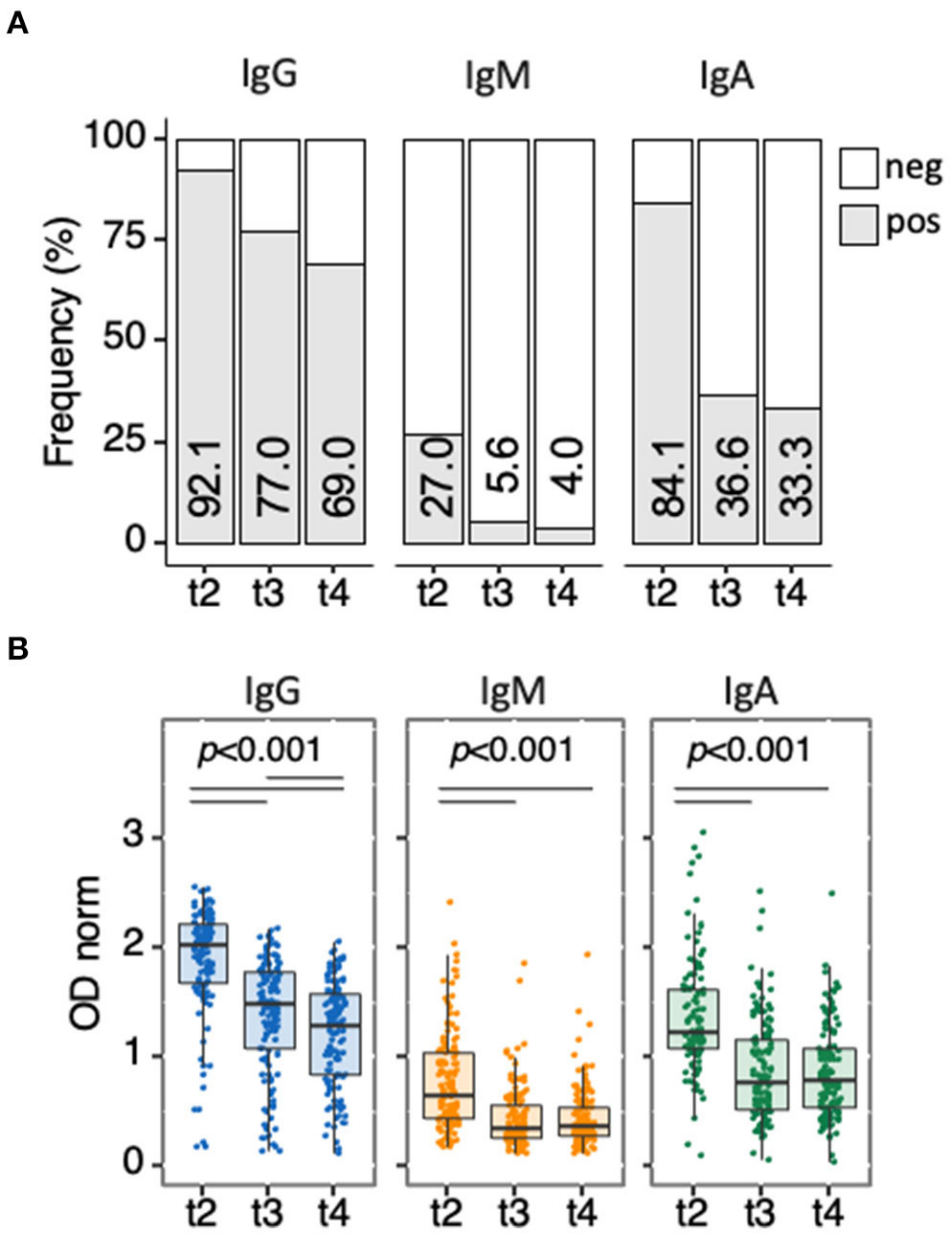
Waning of anti-spike antibody responses along time. Follow-up analysis of antibody responses for anti-spike IgG, IgM, and IgA antibodies in HD patients (*n* = 126) collected at t2, t3, and t4. **(A)** Seroconversion is determined and presented as in Figure 2. Pearson's Chi-squared test (with Yates' continuity correction) was used to determine differences over time. **(B)** anti-spike antibodies in patients, determined and presented as in Figure 3. Differences were determined by the Quade test for antibody levels along time (all 3 panels), the Wilcoxon signed-rank test for pairwise comparison in each panel (*p-*values indicated by horizontal black bars). Detailed *p-*values presented in Supplementary Table 10. t2, sera collected 42 days post-first vaccine dose; t3, sera collected ~140 days post-first vaccine dose; t4, sera collected 180 days post-first vaccine dose.

Waning of the humoral response in HD patients was assessed in 126/143 patients who complied with two additional collections at t3 (140 days after first vaccine dose, corresponding to 4 months post second dose) and t4 (180 days after first vaccine dose, corresponding to 5 months post second dose) (Figure 6, Supplementary Table 10). Anti-spike IgG levels decreased in the 100-day interval between t2 and t3 and during the following 40 days between t3 and t4 (median [IQR]: 2.03 [1.69–2.21] at t2; 1.49 [1.08–1.79] at t3; 1.28 [0.84–1.58] at t4, t2 vs. t3 and t3 vs. t4 *p* < 2 × 10^−16^). IgM and IgA antibodies levels, which were not elevated at t2, decreased between t2 and t3 (*p* < 2 × 10^−16^ for both) and remained at the same low values between t3 and t4 (*p* = 0.750 for IgM and *p* = 0.410 for IgA), confirming IgG is the dominant class of reactive antibodies elicited by BNT162b2 vaccine. Anti-spike IgG waning over time led to a progressive decrease in positivity, resulting in 39/126 (31%) seronegatives at t4.

**Figure 7:**
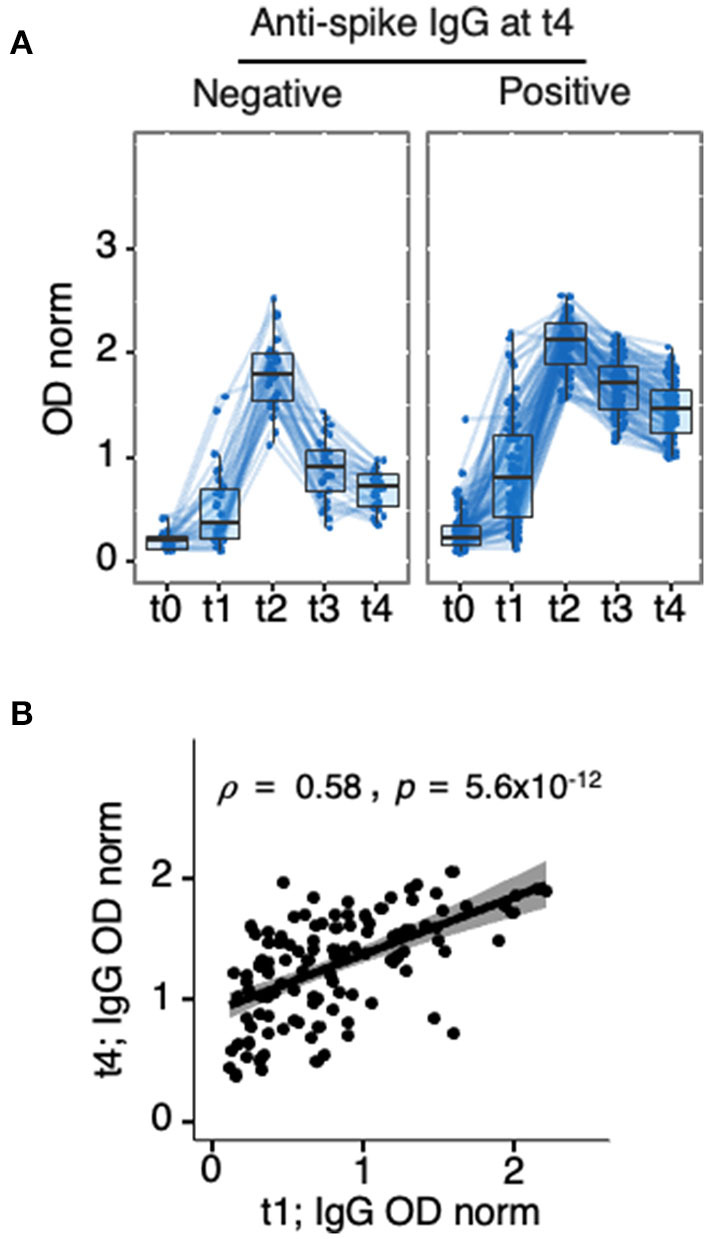
Anti-spike IgG levels at t1 as a predictor of seroreversion. Longitudinal analysis of anti-spike IgG in HD patients who participated in the 5 collection time points (t0, t1, t2, t3, and t4). Ten non-responders (seronegative at t1 and t2) were excluded from the analysis, (*n* = 116) **(A)** Partitioning of data presented in Figures 3, 6, showing HD patients classified as negative (*n* = 39, left), or positive (*n* = 87, right) at t4. **(B)** Spearman correlation coefficients (ρ) analysis of anti-spike IgG levels at t4 vs. t1 in same (*n* = 126) patients, *p-*value inserted in the panel was obtained by Spearman's rank-order non-parametric test. Detailed *p-*values presented in Supplementary Tables 11–13. t0, sera collected on the day of first vaccine dose; t1, sera collected 21 days post-first vaccine dose; t2, sera collected 42 days post-first vaccine dose; t3, sera collected ~140 days post-first vaccine dose; t4, sera collected 180 days post-first vaccine dose.

Of the 39 patients who presented values below the threshold of positivity by t4, 10 were originally non-responders whereas 29 (23% of the cohort) were *bona-fide* seroreverters (Table 2). As for anti-spike seroconversion, anti-spike seroreversion did not correlate with HBV vaccination response, with 11/29 (37.9%) seroreverters and 44/87 (50.6%) seropositive at t4 presenting anti-HBs antibodies *(p* = 0.286). Analysis of anti-spike IgG levels along the 5 time points (Figure 7, Supplementary Tables 11–13) revealed that patients who remained seropositive 5 months post second dose (t4, *n* = 87) presented higher anti-spike IgG levels at earlier time points. This was already evident as early as 3 weeks after the first dose (t1) (0.80 [0.44–1.22] in positive vs. 0.37 [0.24–0.70] in the negative, *p* = 7.03 × 10^−04^). Analysis performed on the 116 participants that either maintained or lost IgG positivity at t4 confirmed IgG levels at earlier time points, including t1, correlated with values at t4 (ρ= 0.58, *p* < 5.6 × 10^−12^ for t4 vs. t1). In agreement with the latter finding, and as for t2, age barely contributed to seroreversion at t4 (*p*= 0.027), and no specific clinical conditions or treatment could explain this outcome (Table 2).

**Table 2:** Clinical characterization of nonresponders, responders who lost (seroreverted), and responders who maintained (seropositive) anti-spike IgG at t4.

**Characteristics**	**Sero-reverted**	**Seropositive**
	**(*N =* 29)**	**(*N =* 87)**
Age (years), median [IQR]	76 [64–84]^*^	69 [59–79]^*^
Sex, men, *n* (%)	19 (65.52)	61 (70.11)
Dialysis duration (months), median [IQR]	46 [23–99]	47 [20–121]
**Comorbidities**		
Charlson Score (age–adjusted), median [IQR]	8 [6–9]	7 [5–8]
Obesity (BMI>30 Kg/m^2^), *n* (%)	11 (37.93)	23 (26.44)
Endocrine diseases (Diabetes mellitus and others), *n* (%)	17 (58.62)	41 (47.13)
Cardiovascular disease, excluding essential hypertension, *n* (%)	16 (55.17)	33 (37.93)
Essential hypertension, *n* (%)	23 (79.31)	64 (73.56)
Congenital or acquired immunodeficiency, *n* (%)	3 (10.34)	3 (3.45)
Chronic pulmonary disease, *n* (%)	8 (27.59)	10 (11.49)
Chronic liver disease, *n* (%)	0	6 (6.90)
Rheumatic disease, *n* (%)	0	5 (5.75)
Cancer in the last 5 years (non-leukemia), *n* (%)	4 (13.79)	16 (18.39)
Past Kidney transplant, *n* (%)	6 (20.69)	13 (14.94)
On immunosuppressive drugs, *n* (%)	1 (3.45)	6 (6.90)

*N, total number of individuals; n, number of individuals with a given event; %, percentage; IQR, interquartile range; t4, sera collected 180 days post-first vaccine dose*.

*Statistical tests to compare sero-reverters with seropositives at t4 were applied according to the type of variable (categorical - Fisher's exact test or continuous—Wilcoxon rank sum test)*.

* *W = 2,042, p-value = 0.0273, 95% CI [1.00–11.00]*.

*All others, not significant*.

## Discussion

In this study, we evidence that while seroconversion following priming was lower in HD patients when compared to age-matched controls, most reach positivity for anti-spike IgG after a second BNT162b2 vaccine dose. Furthermore, waning of the humoral immune response is readily detectable 4 months after the second vaccination. Together, these findings further advocate for the specific management of HD patients during the COVID-19 pandemic.

The original guidelines for BNT162b2 vaccine regimen were of 2 doses administrated at 3–4 weeks interval, although the time between doses has been debated, and more recently, a reinforcing third dose has been approved for specific population subgroups. Our data confirmed that the second dose is essential to reach a high frequency of seroconversion in HD patients, as was shown before in smaller cohorts [e.g., (20), *n* = 22, (21), *n* = 10]. The heterogeneity we evidence in the levels of reactive Ig after a single vaccine dose, with close to 70% seronegative patients, advocates not to extend the interval between the 2 doses to rapidly reach high levels of antibodies. This proposition is consistent with a previous study addressing a very large population which evidenced BNT162b2 vaccine effectiveness was of 57% after one dose and 97% after two doses (11). Other studies argue that extending the time of prime-boost interval enhances the recall response (22), which may be beneficial in the long term, notably by prolonging immune memory. However, in times of pandemic, with actual or risk of high infection incidence, frail populations would benefit from rapidly reaching effective immunity.

In our immunogenicity analysis, we excluded participants who were identified as previously exposed to SARS-CoV-2, so as to evaluate the immune response induced *de novo* by the vaccine. In preexposed individuals, the first vaccine dose acts as a boost (23–26), as confirmed in our enlarged cohort of controls after analyzing specifically the anti-N positive participants at t0 (17), who were excluded in this work. However, some participants may have been preexposed to SARS-CoV-2 and already lost anti-N reactivity by the time the study was initiated (27). It is plausible that the 2 HD participants found anti-spike IgG positive at t0 were preexposed and had already lost anti-N reactivities by the time of our analysis.

One strength of our study is the partitioning of the patient and control cohort by age groups. With this approach, we reveal the difference between HD patients and controls lies in the younger participants. This finding is relevant as a third booster shot has just been approved and has been prioritized by age and specific conditions that do not include HD so far. Analysis upon age partitioning also completes previous studies reporting that COVID-19 vaccines are less efficacious at inducing antibody in HD patients (14, 20, 28). Nevertheless, our analysis confirms aging is a dominant trait affecting mRNA vaccine effectiveness (17, 29). Finally, our finding that the younger group of patients shows lower levels of anti-spike reactivities than age-matched control, and is barely differentiated from aged patients, is compatible with a signature of early immune senescence in this population.

Although IgG is the dominant class of anti-spike antibodies induced upon BNT162b2 vaccination, our results indicate 84% of the patients mounted an IgA response. Secretory IgA, the product of *a bona fide* germinal center reaction, acts at the mucosa, the site of primary SARS-CoV-2 infection, and anti-spike secretory IgA responses with neutralizing capacity were reported following natural SARS-CoV-2 infection (30). Whether the vaccine reactive IgA encompasses secretory IgA remains to be assessed. The IgM levels were relatively low in both cohorts, possibly related to IgM being produced transiently as the result of a rather T-cell independent process. In support of a rather T-cell independent response for both IgA and IgM, the decline in reactivity for these isotypes was severe by t3 in most patients.

Only 42% of patients in the HD cohort presented anti-HBs antibodies following Hepatitis B vaccination. A similar range of hepatitis B vaccination effectiveness was reported previously (31). The lack of correlation between anti-spike Ig levels, or seroconversion, after BNT162b2 vaccination at t2 as at t4 and responsiveness to hepatitis B immunization is in accordance with a previous publication addressing a smaller cohort (*n* = 81) at early time point post SARS-CoV2 vaccination (32). The Hepatitis B vaccine is a subunit vaccine (HBs antigen mixed with adjuvant), while BNT162b2 is an mRNA embedded in lipid nanoparticles. The findings may support that mRNA vaccines present increased immunogenicity when compared with more standard subunit vaccines. Alternatively, spike may be more immunogenic than HBs. Irrespectively of these considerations, serial recall injections are common practice for Hepatitis B vaccine in identified antibody negative individuals. Similarly, serial recall BNT162b2 vaccinations may be required for those individuals identified as poor responders.

After a prime-boost induction phase, vaccine-reactive antibody levels are expected to decrease. However, for most vaccines, long-term immunological memory is associated with detectable antibody reactivities in healthy individuals, albeit at low levels. This is the case for BNT162b2, as 6 months post-second dose a vast majority of 1,370 HCW cohort were still seropositive (33). However, waning vaccine-induced immunity in the general population by around month 4 postvaccination is revealed in countries with high SARS-CoV-2 incidence, a discrepancy likely related to the change in the dominant variant of SARS-CoV-2, which partially escapes immunity induced by the ancestral form of the spike (34). In HD patients, longitudinal studies addressing decreased immunity upon mRNA vaccination are still scarce. It was recently reported that 10/172 HD patients (6%) serorevert by 3 months after the second vaccine dose (35). Another study conducted on 41 HD patients, indicates that seroconversion rate decreases from 98% at 1 month to 66% at 6 months after the second dose (36). In our work, we dissociated non-responders to the 2-dose vaccine regimen from *bona fide* seroreverters, studied a cohort of similar size to that in (35), and a duration approaching that of (36) to reveal 29/145 (20%) lost positivity in the 4 months following the second dose, a result in accordance with the previous studies. We evidenced that levels of specific IgG after the first or second dose can serve as predictors of the persistence of seroconversion, a correlation reported previously in a smaller (*n* = 41) cohort (36). Altogether, our kinetic analyses support the previous proposition of additional booster doses for this group of vulnerable patients (37).

We tested whether seroconversion and seroreversion were correlated with immunosuppressive therapies or with disease duration, severity, or activity, and found only signals of little significance. This result may be related to only few patients being under immunosuppressors in our cohort and most of these under mild therapies, and also to the evidence HD patients are a heterogeneous group in what concerns comorbidities. In concordance with multiple factors modulating humoral immunity, only the age-adjusted Charlson comorbidity index predicted anti-spike IgG levels at t2.

The limitations of our study include that systematic surveillance for SARS-CoV-2 infections was not performed. Larger cohorts of HD patients will be necessary to evaluate vaccine effectiveness in preventing infection, morbidities or death, and viral transmission. The study did not include functional assays such as neutralizing antibodies, which have been shown to be predictive of protection from severe disease and to a lower extent from infection (38). However, levels of anti-spike reactivity elicited by SARS-CoV-2 mRNA vaccines correlate with *in vitro* neutralization of spike-pseudoviruses and SARS-CoV-2, including variants of concern, by us and others (39, 40). Moreover, both binding and neutralizing antibodies correlate with mRNA vaccine efficacy (41, 42). We also did not address cellular immunity. Previous studies in HD patients revealed a strong correlation between anti-spike antibody detection and the frequency or the total number of specific plasmablasts and memory B cells (43) and also with specific T cell responses (21). Others failed to evidence this correlation when comparing HD and controls, with decreased humoral but not T cell responses (44). Detangling such discrepancies will await further analysis and standardized protocols. Similarly to other vaccines, it remains likely that detection of reactive antibodies are positive indicators of the engagement and memory of the adaptive immune system.

Despite these limitations, our findings highlight that HD patients may benefit from tailored COVID-19 vaccination regimens and follow-up. This concern is acute as variants less susceptible to vaccine-induced immunity have replaced worldwide the ancestral virus from which BNT162b2 was derived.

## Data Availability Statement

The original contributions presented in the study are included in the article/Supplementary Materials, further inquiries can be directed to the corresponding author/s.

## Ethics Statement

The studies involving human participants were reviewed and approved by the Ethics Committees of (i) DaVita in Portugal, (ii) Centro Hospitalar Lisboa Ocidental Portugal, and (iii) the Administração Regional de Lisboa e Vale do Tejo Portugal. The patients/participants provided their written informed consent to participate in this study.

## Author Contributions

AW, JD, and CP-G conceived and designed the study, validated the data, and finalized the manuscript. M-LB performed the statistical analysis. LG and IG organized the database. ND wrote the first draft of the manuscript. PB, AB, VM, PM, OA, LK, and MM performed laboratory assays. RA, PC, EN, MP, and AF collected samples and clinical data of the test cohort. TN and JF-V organized the collection of samples and clinical data of the control cohorts. All authors approved the submitted version.

## Funding

This work benefited from COVID-19 emergency funds 2020 from Calouste Gulbenkian Foundation and from Oeiras and Almeirim city councils. It was also supported by the Science and Technology Foundation, Ministry of Education and Science (FCT, Portugal) through Project 754-Research4COVID-19-−2nd edition, by PORLisboa 2020, Portugal 2020 and European Union, through European Regional Development Fund through the project FEDER/072558, and from research funds from DaVita, Portugal. The funding entities had no role in study design, data collection and analysis, or decision to publish.

## Conflict of Interest

The authors declare that the research was conducted in the absence of any commercial or financial relationships that could be construed as a potential conflict of interest.

## Publisher's Note

All claims expressed in this article are solely those of the authors and do not necessarily represent those of their affiliated organizations, or those of the publisher, the editors and the reviewers. Any product that may be evaluated in this article, or claim that may be made by its manufacturer, is not guaranteed or endorsed by the publisher.
