# Supplementary material for: Longitudinal Analysis of Antibody Responses to the mRNA BNT162b2 Vaccine in Patients Undergoing Maintenance Hemodialysis: A 6-Month Follow-Up
Source: Front Med (Lausanne). 2021 Dec 24;8:796676. doi: 10.3389/fmed.2021.796676 (PMC8740691; doi:10.3389/fmed.2021.796676)
Supplement: Supplementary file 1 [file Table_1.pdf]

Supplementary Table 1. Comparison of anti-Spike IgG, IgM, and IgA positivity at t1 vs t2 (data presented in Figure 2).

				Positivity; n (%; 95% CI)		
	**Isotype**	**Age**	**n**	**t1**	**t2**	***p*-value***
Patients	IgG	All	143	42 (29.4; 22.5-37.3)	130 (90.9; 85.1-94.6)	8.01×10^{-26}
		27-70	66	22 (33.3; 23.2-45.3)	63 (95.5; 87.5-98.4)	1.19×10^{-12}
		71-93	77	20 (26.0; 17.5-36.7)	67 (87.0; 77.7-92.8)	7.61×10^{-14}
	IgM	All	143	17 (11.9; 7.6-18.2)	42 (29.4; 22.5-37.3)	0.0005
	IgA	All	143	59 (41.3; 33.5-49.5)	120 (83.9;77.0-89.0)	2.27×10^{-13}
Controls	IgG	All	143	71 (49.7; 41.6-57.7)	136 (95.1; 90.2-97.6)	2.59×10^{-17}
		30-70	66	48 (72.7; 61.0-82.0)	65 (98.5; 91.9-99.7)	7.27×10^{-05}
		71-96	77	23 (29.9; 20.8-40.8)	71 (92.2; 84.0-96.4)	8.08×10^{-15}
	IgM	All	143	22 (15.4; 10.4-22.2)	36 (25.2;18.8-32.9)	0.0559
	IgA	All	143	72 (50.4; 42.2-58.4)	113 (79.0;71.6-84.9)	7.47×10^{-07}

n, number of individuals with a given event; %, percentage; CI, confidence interval; t1 – sera collected 21 days post-1st vaccine dose; t2 – sera collected 42 days post-1st vaccine dose.

*Chi squared test with BH method for *p*-value adjustment used to compare positivity between t1 and t2.
