# Supplementary material for: Longitudinal Analysis of Antibody Responses to the mRNA BNT162b2 Vaccine in Patients Undergoing Maintenance Hemodialysis: A 6-Month Follow-Up
Source: Front Med (Lausanne). 2021 Dec 24;8:796676. doi: 10.3389/fmed.2021.796676 (PMC8740691; doi:10.3389/fmed.2021.796676)
Supplement: Supplementary file 3 [file Table_2.pdf]

Supplementary Table 2. Comparison of anti-Spike IgG, IgM, and IgA positivity at in patients and controls, according to time-point (data presented in Figure 2).

	p*-value	
**Isotype**	**t1**	**t2**
IgG	0.0014	0.2463
IgM	0.5068	0.5068
IgA	0.3088	0.3612

t1 – sera collected 21 days post-1st vaccine dose; t2 – sera collected 42 days post-1st vaccine dose.
*Chi squared test with BH method for *p*-value adjustment used to compare positivity between patients and controls.
