# Supplementary material for: Longitudinal Analysis of Antibody Responses to the mRNA BNT162b2 Vaccine in Patients Undergoing Maintenance Hemodialysis: A 6-Month Follow-Up
Source: Front Med (Lausanne). 2021 Dec 24;8:796676. doi: 10.3389/fmed.2021.796676 (PMC8740691; doi:10.3389/fmed.2021.796676)
Supplement: Supplementary file 4 [file Table_3.pdf]

Supplementary Table 3. IgG, IgM, and IgA levels at t0, t1, and t2, according to age groups (data presented in Figure 3).

				Median [IQR]		
	**Isotype**	**Age**	**n**	**t0**	**t1**	**t2**
Patients	IgG	All	143	0.22 [0.16-0.32]	0.63 [0.32-1.08]	2.05 [1.67-2.23]
		27-70	66	0.22 [0.15-0.32]	0.68 [0.45-1.21]	2.11 [1.87-2.28]
		71-93	77	0.22 [0.17-0.30]	0.47 [0.28-1.00]	1.87 [1.57-2.18]
	IgM	27-93	143	0.33 [0.24-0.56]	0.49 [0.32-0.75]	0.66 [0.45-1.07]
	IgA	27-93	143	0.53 [0.35-0.73]	0.85 [0.63-1.10]	1.22 [1.10-1.63]
Controls	IgG	All	143	0.19 [0.15-0.24]	0.96 [0.46-1.39]	1.82 [1.70-1.90]
		30-70	66	0.17 [0.14-0.21]	1.27 [0.93-1.49]	1.81 [1.72-1.89]
		71-96	77	0.21 [0.16-0.26]	0.52 [0.38-1.21]	1.83 [1.66-1.92]
	IgM	30-96	143	0.49 [0.34-0.66]	0.49 [0.35-0.75]	0.56 [0.35-1.01]
	IgA	30-96	143	0.56 [0.38-0.80]	1.00 [0.69-1.16]	1.15 [1.04-1.35]

n, number of individuals with a given event; IQR, interquartile range; t0 – sera collected on day of 1st vaccine dose; t1 – sera collected 21 days post-1st vaccine dose; t2 – sera collected 42 days post-1st vaccine dose.
