# Supplementary material for: Longitudinal Analysis of Antibody Responses to the mRNA BNT162b2 Vaccine in Patients Undergoing Maintenance Hemodialysis: A 6-Month Follow-Up
Source: Front Med (Lausanne). 2021 Dec 24;8:796676. doi: 10.3389/fmed.2021.796676 (PMC8740691; doi:10.3389/fmed.2021.796676)
Supplement: Supplementary file 5 [file Table_4.pdf]

Supplementary Table 4. Comparison of IgG, IgM, and IgA levels at t0, t1, and t2 between HD patients and controls, in individuals above and below 70 years (data presented in Figure 3).

		p*-value		
**Isotype**	**Age**	**t0**	**t1**	**t2**
IgG	All	0.0103	4.98×10^{-04}	5.37×10^{-06}
	27-70y	0.0170	2.01×10^{-05}	5.72×10^{-09}
	71-96y	0.2941	0.2492	0.2492
IgM	All	1.75×10^{-05}	0.3917	0.0913
	27-70y	0.0336	0.0161	0.7138
	71-96y	0.0003	0.3009	0.0074
IgA	All	3.30×10^{-16}	0.5577	3.30×10^{-16}
	27-70y	0.4218	0.0219	0.0219
	71-96y	0.1445	0.9665	0.2426

t0 – sera collected on day of 1st vaccine dose; t1 – sera collected 21 days post-1st vaccine dose; t2 – sera collected 42 days post-1st vaccine dose.
*Wilcoxon rank sum test with BH method for *p*-value adjustment was used to compare Ig levels between patients and controls.
