# Supplementary material for: Longitudinal Analysis of Antibody Responses to the mRNA BNT162b2 Vaccine in Patients Undergoing Maintenance Hemodialysis: A 6-Month Follow-Up
Source: Front Med (Lausanne). 2021 Dec 24;8:796676. doi: 10.3389/fmed.2021.796676 (PMC8740691; doi:10.3389/fmed.2021.796676)
Supplement: Supplementary file 6 [file Table_5.pdf]

Supplementary Table 5. Comparison of IgG, IgM, and IgA levels in individuals above or below 70 years old in each time-point (data presented in Figure 3).

		p*-value		
	**Isotype**	**t0**	**t1**	**t2**
Patients	IgG	0.6403	0.0503	0.0115
	IgM	0.0006	0.0006	0.0001
	IgA	0.8318	0.8318	0.8318
Controls	IgG	0.0023	5.09×10^{-07}	0.8380
	IgM	0.0016	2.66×10^{-13}	1.10×10^{-10}
	IgA	9.73×10^{-04}	5.28×10^{-08}	6.60×10^{-16}

t0 – sera collected on day of 1st vaccine dose; t1 – sera collected 21 days post-1st vaccine dose; t2 – sera collected 42 days post-1st vaccine dose.
*Wilcoxon rank sum test with BH method for *p*-value adjustment was used to compare Ig levels between age groups.
