# Supplementary material for: Longitudinal Analysis of Antibody Responses to the mRNA BNT162b2 Vaccine in Patients Undergoing Maintenance Hemodialysis: A 6-Month Follow-Up
Source: Front Med (Lausanne). 2021 Dec 24;8:796676. doi: 10.3389/fmed.2021.796676 (PMC8740691; doi:10.3389/fmed.2021.796676)
Supplement: Supplementary file 7 [file Table_6.pdf]

Supplementary Table 6. *p*-values comparing IgG, IgM, and IgA levels over time in HD patients and controls (data presented in Figure 3).

p*-value			**All**		**27-70y**		**71-96y**	
	**Isotype**	**Time**	**t0**	**t1**	**t0**	**t1**	**t0**	**t1**
Patients	**IgG**	t1	$2.2x10^{-16}$	-	$6.2x10^{-12}$	-	$2.7x10^{-10}$	-
		t2	$2.2x10^{-16}$	$2.2x10^{-16}$	$2.0x10^{-12}$	$2.0x10^{-12}$	$5.5x10^{-14}$	$5.5x10^{-14}$
Controls		t1	$2.2x10^{-16}$	-	$2.0x10^{-12}$	-	$7.5\ x10^{-13}$	-
		t2	$2.2x10^{-16}$	$2.2x10^{-16}$	$2.0x10^{-12}$	$2.0x10^{-12}$	$4.4\ x10^{-14}$	$4.4\ x10^{-14}$
Patients	IgM	t1	$8.7x10^{-13}$	-	$9.3x10^{-6}$	-	$1.9x10^{-8}$	-
		t2	$2.2x10^{-16}$	$4.5x10^{-15}$	$7.1x10^{-12}$	$1.7x10^{-8}$	$2.3x10^{-11}$	$8.8x10^{-8}$
Controls		t1	0.0602	-	$1.3x10^{-8}$	-	0.0004	-
		t2	0.00005	0.0405	$1.1x10^{-8}$	0.07163	0.3502	0.2372
Patients	IgA	t1	$2.2x10^{-16}$	-	$1.8x10^{-11}$	-	$1.4x10^{-12}$	-
		t2	$2.2x10^{-16}$	$2.2x10^{-16}$	$3.4x10^{-12}$	$1.0x10^{-10}$	$1.1x10^{-13}$	$7.8x10^{-13}$
Controls		t1	$3.3x10^{-16}$	-	$3.2x10^{-12}$	-	$3.2x10^{-6}$	-
		t2	$3.3x10^{-16}$	$2.3x10^{-11}$	$3.2x10^{-12}$	0.0181	$1.3x10^{-12}$	$5.4x10^{-10}$

t0 – sera collected on day of 1st vaccine dose; t1 – sera collected 21 days post-1st vaccine dose.
*Pairwise Wilcoxon signed-rank test was used to compare Ig levels between time points.
