# Supplementary material for: Longitudinal Analysis of Antibody Responses to the mRNA BNT162b2 Vaccine in Patients Undergoing Maintenance Hemodialysis: A 6-Month Follow-Up
Source: Front Med (Lausanne). 2021 Dec 24;8:796676. doi: 10.3389/fmed.2021.796676 (PMC8740691; doi:10.3389/fmed.2021.796676)
Supplement: Supplementary file 8 [file Table_7.pdf]

Supplementary Table 7. Comparison of global differences of IgG, IgM, and IgA levels over time in HD patients and controls, according to age groups (data presented in Figure 3).

***p*-value†**		**All**	**27-70y**	**71-96y**
Patients	IgG	$2.2x10^{-16}$	$2.2x10^{-16}$	$2.2x10^{-16}$
	IgM	$2.2x10^{-16}$	$8.5x10^{-13}$	$2.2x10^{-16}$
	IgA	$2.2x10^{-16}$	$2.2x10^{-16}$	$2.2x10^{-16}$
Controls	IgG	$2.2x10^{-16}$	$2.2x10^{-16}$	$2.2x10^{-16}$
	IgM	9.164e-06	$2.2x10^{-16}$	0.02799
	IgA	$2.2x10^{-16}$	$2.2x10^{-16}$	$2.2x10^{-16}$

†Quade test was used to test for group differences over time.
