# Supplementary material for: Longitudinal Analysis of Antibody Responses to the mRNA BNT162b2 Vaccine in Patients Undergoing Maintenance Hemodialysis: A 6-Month Follow-Up
Source: Front Med (Lausanne). 2021 Dec 24;8:796676. doi: 10.3389/fmed.2021.796676 (PMC8740691; doi:10.3389/fmed.2021.796676)
Supplement: Supplementary file 9 [file Table_8.pdf]

Supplementary Table 8. Anti-spike IgG levels in HD patients, treated or not with immunosuppressors (data presented in Figure 4).

			Median [IQR]		
	**Age**	**n**	**t0**	**t1**	**t2**
Immuno-suppression	42-63y	11	0.16 [0.13-0.25]	0.37 [0.19-0.59]	1.85 [1.27-2.17]
No immuno-suppression	42-69y	50	0.23 [0.15-0.34]	0.78 [0.57-1.21]	2.11 [1.94-2.26]
p-value* (treated vs controls)			0.1798	0.0127	0.0969

n, number of individuals with a given event; IQR, interquartile range; t0 – sera collected on day of 1st vaccine dose; t1 – sera collected 21 days post-1st vaccine dose; t2 – sera collected 42 days post-1st vaccine dose.
*Wilcoxon rank sum test with BH method for *p*-value adjustment was used to compare Ig levels between patients treated or not with immunosuppressors.
