# Supplementary material for: Longitudinal Analysis of Antibody Responses to the mRNA BNT162b2 Vaccine in Patients Undergoing Maintenance Hemodialysis: A 6-Month Follow-Up
Source: Front Med (Lausanne). 2021 Dec 24;8:796676. doi: 10.3389/fmed.2021.796676 (PMC8740691; doi:10.3389/fmed.2021.796676)
Supplement: Supplementary file 10 [file Table_9.pdf]

Supplementary Table 9. *p*-values comparing IgG levels over time in HD patients, treated or not with immunosuppressors (Figure 4).

p*-value	**Time**	**t0**	**t1**
Immuno-suppression	t1	0.1014	-
	t2	0.0006	0.0048
No immuno-suppression	t1	$1.9x10^{-12}$	-
	t2	$2.0x10^{-16}$	$7.1x10^{-16}$

t0 – sera collected on day of 1st vaccine dose; t1 – sera collected 21 days post-1st vaccine dose.
*Pairwise Wilcoxon signed-rank test was used to compare Ig levels between time points
