# Supplementary material for: Longitudinal Analysis of Antibody Responses to the mRNA BNT162b2 Vaccine in Patients Undergoing Maintenance Hemodialysis: A 6-Month Follow-Up
Source: Front Med (Lausanne). 2021 Dec 24;8:796676. doi: 10.3389/fmed.2021.796676 (PMC8740691; doi:10.3389/fmed.2021.796676)
Supplement: Supplementary file 11 [file Table_10.pdf]

Supplementary Table 10. Positivity, Median and Interquartile range [IQR] for anti-spike IgG, IgM, and IgA (data presented in Figure 6).

	**t2 (N=126)**	**t3 (N=126)**	**t4 (N=126)**	***p*-value* t2 vs t3**	***p*-value* t2 vs t4**	***p*-value* t3 vs t4**
anti-spike IgG						
positive, n (%)	116 (92.06)	97 (76.98)	87 (69.05)	0.0017	$8.32x10^{-6}$	0.2015
ODnorm, median [IQR]	2.03 [1.69-2.21]	1.49 [1.08-1.79]	1.28 [0.84-1.58]	$<2x10^{-16}$	$<2x10^{-16}$	$<2x10^{-16}$
anti-spike IgM						
positive, n (%)	34 (26.98)	7 (5.56)	5 (3.97)	$1.365x10^{-5}$	$3.23x10^{-6}$	0.7674
ODnorm, median [IQR]	0.65 [0.43-1.04]	0.34 [0.26-0.56]	0.36 [0.27-0.54]	$<2x10^{-16}$	$<2x10^{-16}$	0.7500
anti-spike IgA						
positive, n (%)	106 (84.13)	46 (36.51)	42 (33.33)	$4.55x10^{-14}$	$2.27x10^{-15}$	0.6918
ODnorm, median [IQR]	1.21 [1.08-1.62]	0.76 [0.52-1.17]	0.78 [0.53-1.08]	$<2x10^{-16}$	$<2x10^{-16}$	0.4100

N, total number of individuals; n, number of individuals with a given event; IQR, interquartile range; t2 – sera collected 42 days post-1st vaccine dose; t3 - sera collected ~140 days post-1st vaccine dose; t4 - sera collected 180 days post-1st vaccine dose. Quade test for differences in anti-spike IgG, IgM, and IgA levels over time, $p<2x10^{-16}$.

*Statistical tests were applied according to the type of variable (Chi-square for positivity and pairwise Wilcoxon signed-rank test for ODnorm).
