# Supplementary material for: Longitudinal Analysis of Antibody Responses to the mRNA BNT162b2 Vaccine in Patients Undergoing Maintenance Hemodialysis: A 6-Month Follow-Up
Source: Front Med (Lausanne). 2021 Dec 24;8:796676. doi: 10.3389/fmed.2021.796676 (PMC8740691; doi:10.3389/fmed.2021.796676)
Supplement: Supplementary file 12 [file Table_11.pdf]

Supplementary Table 11. Comparison of IgG levels between seronegative and seropositive at t4, at indicated time points (data presented in Figure 7A).

	Median [IQR]		
**Time**	**Seronegative n=29**	**Seropositive n=87**	***p*-value***
t0	0.22 [0.14-0.26]	0.22 [0.17-0.36]	$7.79x10^{-02}$
t1	0.37 [0.24-0.70]	0.80 [0.44-1.22]	$7.03x10^{-04}$
t2	1.79 [1.55-1.99]	2.12 [1.89-2.29]	$2.16x10^{-05}$
t3	0.91 [0.69-1.08]	1.71 [1.47-1.87]	$2.48x10^{-14}$
t4	0.72 [0.55-0.84]	1.47 [1.24-1.66]	$4.49x10^{-15}$

t0 – sera collected on day of 1st vaccine dose; t1 – sera collected 21 days post-1st vaccine dose; t2 – sera collected 42 days post-1st vaccine dose; t3 - sera collected ~140 days post-1st vaccine dose; t4 - sera collected 180 days post-1st vaccine dose.
*Wilcoxon rank sum test with BH method for *p*-value adjustment was used to compare Ig levels between patients treated or not with immunosuppressors.
