# Supplementary material for: Longitudinal Analysis of Antibody Responses to the mRNA BNT162b2 Vaccine in Patients Undergoing Maintenance Hemodialysis: A 6-Month Follow-Up
Source: Front Med (Lausanne). 2021 Dec 24;8:796676. doi: 10.3389/fmed.2021.796676 (PMC8740691; doi:10.3389/fmed.2021.796676)
Supplement: Supplementary file 13 [file Table_12.pdf]

**Supplementary Table 12.** *p*-values comparing IgG levels over time in seronegative and seropositive at t4 and (Figure 7A).

| | | ***p*-value*** | | | |
|---|---|---|---|---|---|
| | **Time** | **t0** | **t1** | **t2** | **t3** |
| Seronegative n=29 | t1 | $3.0x10^{-05}$ | - | - | - |
| | t2 | $6.2x10^{-09}$ | $6.2x10^{-09}$ | - | - |
| | t3 | $6.2x10^{-09}$ | $8.6x10^{-05}$ | $6.2x10^{-09}$ | - |
| | t4 | $6.2x10^{-09}$ | 0.0035 | $6.2x10^{-09}$ | $2.3x10^{-05}$ |
| Seropositive n=87 | t1 | $2.4x10^{-15}$ | - | - | - |
| | t2 | $1.\ 4x10^{-15}$ | $1.24x10^{-15}$ | - | - |
| | t3 | $1.24x10^{-15}$ | $1.94x10^{-15}$ | $6.34x10^{-15}$ | - |
| | t4 | $1.24x10^{-15}$ | $2.64x10^{-14}$ | $1.24x10^{-15}$ | $1.94x10^{-15}$ |

t0 – sera collected on day of 1st vaccine dose; t1 – sera collected 21 days post-1st vaccine dose; t2 – sera collected 42 days post-1st vaccine dose; t3 - sera collected ~140 days post-1st vaccine dose; t4 - sera collected 180 days post-1st vaccine dose.
*Pairwise Wilcoxon signed-rank test was used to compare Ig levels between time points. Quade test for differences in anti-spike IgG levels over time, $p<2x10^{-16}$.
