# Supplementary material for: Longitudinal Analysis of Antibody Responses to the mRNA BNT162b2 Vaccine in Patients Undergoing Maintenance Hemodialysis: A 6-Month Follow-Up
Source: Front Med (Lausanne). 2021 Dec 24;8:796676. doi: 10.3389/fmed.2021.796676 (PMC8740691; doi:10.3389/fmed.2021.796676)
Supplement: Supplementary file 14 [file Table_13.pdf]

Supplementary Table 13. Spearman correlation analysis of anti-spike IgG levels at t4 versus IgG levels at earlier collection time points (data for t4 vs t1 presented in Figure 7B).

	Correlation analysis (n=116)		
IgG OD norm	Age Group	*p*-value*	*ρ*†
t4 vs t1	All	$5.623x10^{-12}$	0.5845
	27-70	$7.993x10^{-07}$	0.6213
	71-93	$2.193x10^{-05}$	0.5214
t4 vs t2	All	$5.749x10^{-09}$	0.5082
	27-70	$3.593x10^{-05}$	0.5334
	71-93	$8.605x10^{-05}$	0.4866
t4 vs t3	All	$<2.2x10^{-16}$	0.9426
	27-70	$<2.2x10^{-16}$	0.9331
	71-93	$<2.2x10^{-16}$	0.9444

t0 – sera collected on day of 1st vaccine dose; t1 – sera collected 21 days post-1st vaccine dose; t2 – sera collected 42 days post-1st vaccine dose; t3 - sera collected ~140 days post-1st vaccine dose; t4 - sera collected 180 days post-1st vaccine dose.
* Spearman correlation *p*-value
† Spearman correlation coefficient
