# Supplementary material for: Longitudinal Analysis of Antibody Responses to the mRNA BNT162b2 Vaccine in Patients Undergoing Maintenance Hemodialysis: A 6-Month Follow-Up
Source: Front Med (Lausanne). 2021 Dec 24;8:796676. doi: 10.3389/fmed.2021.796676 (PMC8740691; doi:10.3389/fmed.2021.796676)
Supplement: Supplementary file 15 [file Presentation_1.pdf]

Supplementary References
software for data management, graphical design and statistical analysis

- R, version 4.0.4 GUI 1.74

(R Core Team (2021). R: A language and environment for statisticalcomputing. R Foundation for Statistical Computing, Vienna, Austria. URL https://www.R-project.org/.)

- RStudio, version 1.1.463

(*RStudio Team (2020). RStudio: Integrated Development for R. RStudio, PBC, Boston, MA URL http://www.rstudio.com/.),*

R packages

- Tidyverse

(Wickham et al., (2019). Welcome to the tidyverse. Journal of Open Source Software, 4(43), 1686, https://doi.org/10.21105/joss.01686

- ggplot2

H. Wickham. ggplot2: Elegant Graphics for Data Analysis. Springer-Verlag New York, 2016.

- openxlsx

Philipp Schauberger and Alexander Walker (2020). openxlsx: Read, Write and Edit xlsx Files. R package version 4.2.3. https://CRAN.R-project.org/package=openxlsx

- writexl

Jeroen Ooms (2020). writexl: Export Data Frames to Excel' xlsx' Format. R package version 1.3.1. https://CRAN.R-project.org/package=writexl

- officer

David Gohel (2021). officer: Manipulation of Microsoft Word and PowerPoint Documents. R package version 0.4.0. https://CRAN.R-project.org/package=officer

- rvg

David Gohel (2020). rvg: R Graphics Devices for Vector Graphics Output. R package version 0.2.5. https://CRAN.R-project.org/package=rvg

- ggpubr

Alboukadel Kassambara (2020). ggpubr: 'ggplot2' Based Publication Ready Plots. R package version 0.4.0. https://CRAN.R-project.org/package=ggpubr
