# Supplementary material for: Longitudinal Analysis of Antibody Responses to the mRNA BNT162b2 Vaccine in Patients Undergoing Maintenance Hemodialysis: A 6-Month Follow-Up
Source: Front Med (Lausanne). 2021 Dec 24;8:796676. doi: 10.3389/fmed.2021.796676 (PMC8740691; doi:10.3389/fmed.2021.796676)
Supplement: Supplementary file 16 [file Image_1.pdf]

Supplementary Figure S1

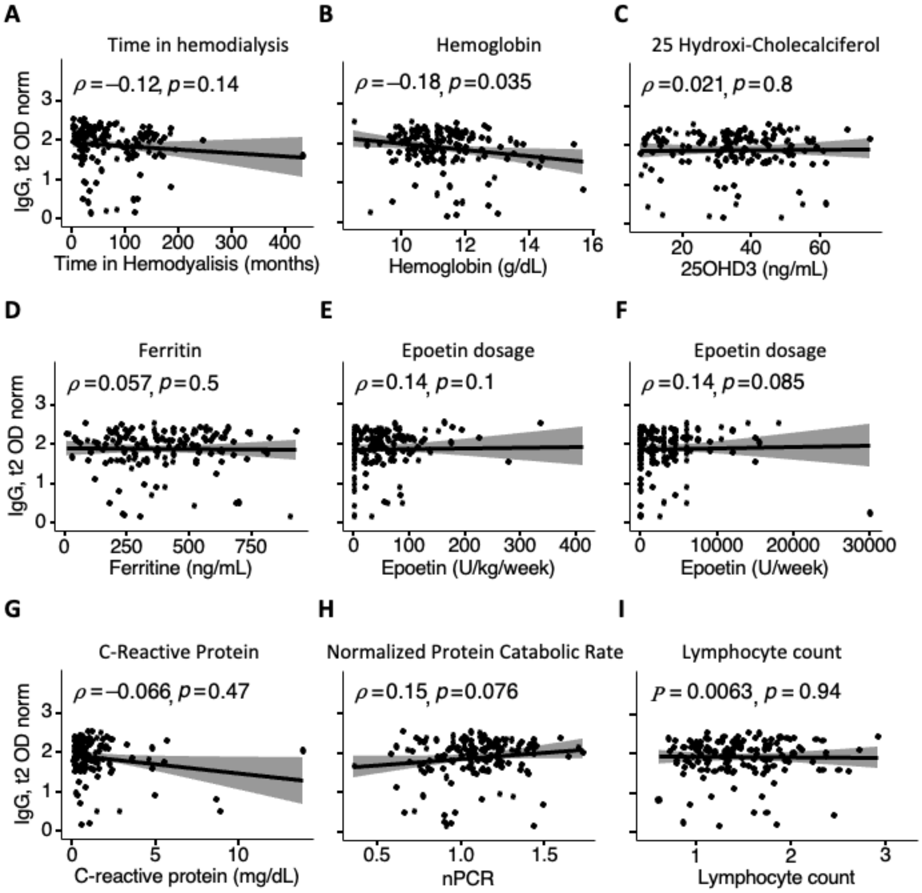

Supplementary Figure S1. Spearman rank correlation coefficient analysis of anti-Spike IgG at t2 with relevant pathology indicators (n=142). Correlation with time in hemodialysis (A); with Hemoglobin levels (B); with 25 hydroxi-cholecalciferol (C); with Ferritin (D); with Epoetin/Kg (E) and Epoetin/U per week (F); with C-reactive protein (G); with normalized Protein Catabolic Rate (nPCR) (H); and with peripheral blood lymphocyte count (x10^{-9}/L) (I). In panel (I) 2 outliers were excluded for abnormally high counts (9 and 50 x10^{-9}/L), both from patients diagnosed with leukemia.
